# Toll-Like Receptor and Accessory Molecule mRNA Expression in Humans and Mice as Well as in Murine Autoimmunity, Transient Inflammation, and Progressive Fibrosis

**DOI:** 10.3390/ijms140713213

**Published:** 2013-06-26

**Authors:** Santhosh Kumar Vankayala Ramaiah, Roman Günthner, Maciej Lech, Hans-Joachim Anders

**Affiliations:** Medical Clinic and Policlinic IV, Nephrological Center, University of Munich, Munich 80336, Germany; E-Mails: santosh.kumar@med.uni-muenchen.de (S.K.V.R.); roman.guenthner@med.uni-muenchen.de (R.G.); maciej.lech@med.uni-muenchen.de (M.L.)

**Keywords:** inflammation, Toll-like receptors, infection, fibrogenesis, atrophy, pattern recognition receptors, chronic disease, signalling

## Abstract

The cell type-, organ-, and species-specific expression of the Toll-like receptors (TLRs) are well described, but little is known about the respective expression profiles of their accessory molecules. We therefore determined the mRNA expression levels of LBP, MD2, CD36, CD14, granulin, HMGB1, LL37, GRP94, UNC93b1, TRIL, PRAT4A, AP3B1, AEP and the respective TLRs in human and mouse solid organs. Humans and mice displayed significant differences between their respective mRNA expression patterns of these factors. In addition, the expression profiles in transient tissue inflammation upon renal ischemia-reperfusion injury, in spleens and kidneys from mice with lupus-like systemic autoimmunity, and in progressive tissue fibrosis upon unilateral ureteral obstruction were studied. Several TLR co-factors were specifically regulated during the different phases of these disease entities, suggesting a functional involvement in the disease process. Thus, the organ- and species-specific expression patterns need to be considered in the design and interpretation of studies related to TLR-mediated innate immunity, which seems to be involved in the tissue injury phase, in the phase of tissue regeneration, and in progressive tissue remodelling.

## 1. Introduction

Toll-like receptors (TLR) are germ-line encoded pattern recognition receptors (PRR) of the innate immune system that translate the recognition of pathogen-associated molecular patterns (PAMPs) and tissue damage-associated molecular patterns (DAMPs) into an immediate and antigen-unspecific inflammatory response [1]. Most TLRs localize to the outer plasma membrane, which allows them to recognize PAMPs and DAMPs that are exposed within the extracellular space, such as bacterial cell wall components or releases from necrotic cells [1]. In contrast, the nucleic acid-specific TLRs localize to intracellular endosomes, because nucleic acids are rarely exposed to the outer plasma membrane and rather get released only upon lysis of ingested bacteria or viruses [1]. However, in systemic lupus erythematosus (SLE), endogenous nucleic acids within immune complexes containing nuclear particles reach endosomes via FcR-mediated phagocytosis before they can interact with TLR7 and TLR9 [2,3]. This mechanism illustrates that accessory molecules such as IgG and FcR modulate TLR agonist-TLR interaction, independent from the intracellular TLR-associated adaptor molecules and downstream signalling elements.

Meanwhile, several such accessory co-factors of TLR activation have been described [4]. Lipopolysaccharide (LPS)-binding protein (LBP) is a 481-amino acid soluble acute phase protein that binds to several cell wall components from gram positive and gram negative bacteria and promotes their interaction with CD14, which is needed for these components to activate TLR4, but also TLR1, TLR2, and TLR6 [5,6]. MD2 is a 160-amino acid glycosylated soluble protein that binds to the inner side of the horseshoe-shaped extracellular domain of TLR4 [7]. MD2 is required for LPS-mediated activation of TLR4 as it links the lipid chains of LPS to TLR4’s recognition site and two MD2 molecules are needed for the homo-dimerization of TLR4 [8]. CD36 is a 472-amino acid double membrane-spanning glycoprotein of the scavenger receptor family that enhances innate immune activation to some but not all TLR2-TLR6 ligands [9]. CD36-mediated ligand discrimination also refers to endogenous ligands such as oxidized LDL and beta-amyloid fibrils at TLR4-TLR6. CD36 shapes ligand specificities of TLR heterodimers in lipid rafts [10]. CD14 is a 375-amino acid soluble glycoprotein that is also present as a GPI-anchored membrane protein on myeloid cells. CD14 has an unusual capacity to interact with multiple TLR ligands and to enhance their capacity to activate TLRs, but the nature of this promiscuous ligand interaction and co-factor function is yet unclear [11,12]. The transmembrane protein TLR4 interactor with leucine-rich repeats (TRIL) acts similar to CD14 but only on TLR3 and TLR4 [13,14]. Granulin (GRN) is a cysteine-rich glycosylated protein that is activated by proteinase 3 and elastase [15]. Granulin binds to nucleic acids that interact with TLR9 and enhances their activity, e.g., by increasing their endosomal delivery [16]. Similar functions are shared by the 215-amino acid nuclear DNA-binding protein high-mobility group box 1 (HMGB1) and the 37 amino acid amphipathic peptide, LL37, that act as co-factors for the DNA recognition of TLR9 and the RNA recognition of TLR7, respectively [17,18]. A number of intracellular proteins shape TLR activity. Glucose-regulated protein (GRP) 94 and protein-associated-with-TLR4-A (PRAT4A) are intracellular chaperonins that regulate the maturation and folding of all TLRs (except for TLR3) within the endoplasmic reticulum [19]. Uncoordinated 93 homolog B1 (UNC93B1) rather shuttles TLR3, TLR7, TLR8, and TLR9 from the ER to endosomes [20]. Adaptor protein 3 (AP3) specifically traffics TLR9 from the ER to endosomes [21]. Asparagine endopeptidase (AEP) is a lysosomal protease that cleaves TLR9, which is needed for full TLR9 bioactivity [22].

As expression patterns of the TLRs differ among species, we hypothesized the same for their accessory molecules and, hence, determined their mRNA expression profiles in human and mouse organs or during transient as well as progressive sterile inflammation.

## 2. Results and Discussion

### 2.1. TLR Accessory Molecule mRNA Expression in Adult Human Tissues

**Figure 1:**
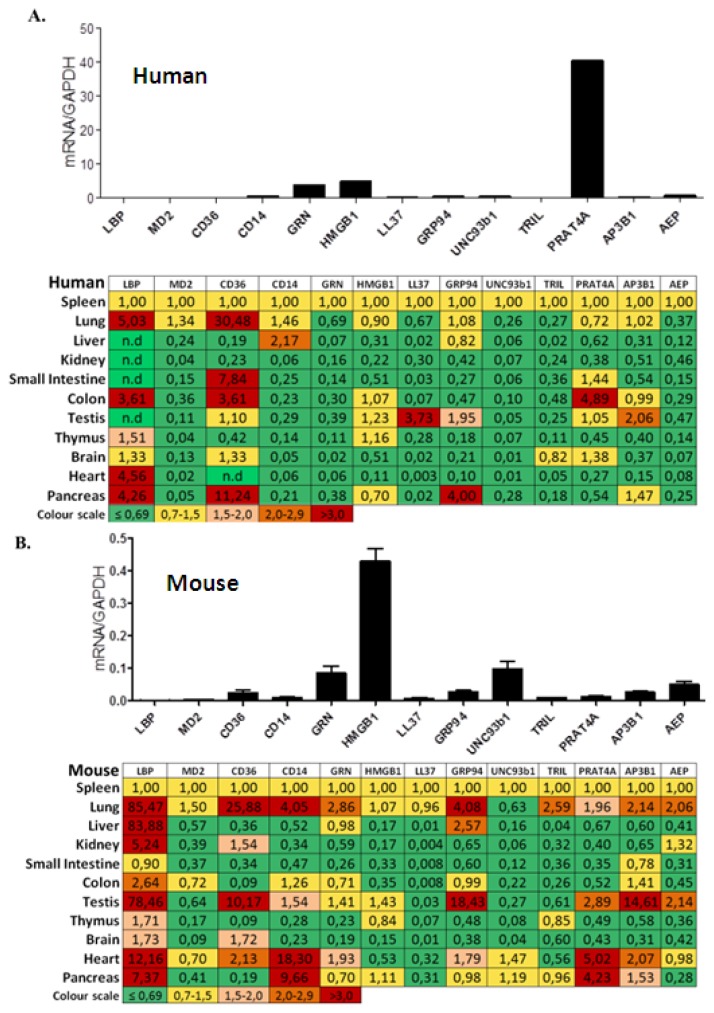
TLR accessory molecular mRNA expression in adult human and mouse tissues. (**A**) Pools of healthy human tissue pre-normalized cDNAs derived from poly(A)-selected DNase-treated RNAs was purified as described in methods. Quantitative real-time PCR analysis was performed and mRNA expression levels of all the organs were normalized to GAPDH mRNA expression level and spleen mRNA levels were illustrated in the form of histograms. The mRNA expression levels of other organs were normalized to spleen. The table displays red to green shades higher or lower relative mRNA expression levels, respectively; (**B**) cDNAs derived from 5 adult 10–12 weeks old C57BL/6 mice in the same manner.

We used real time qRT-PCR to quantify the mRNA expression levels of the following TLR accessory molecules in human solid organs: LBP, MD2, CD36, CD14, GRN, HMGB1, LL37, GRP94, UNC93, TRIL, PRAT4A, AP3B1 and AEP. All of these molecules were constitutively expressed in human spleen but the mRNA expression levels of LBP, MD2, CD36, LL37 and TRIL were low (Figure 1A). LBP expression was higher in lung, colon, heart and pancreas but remained low in liver, kidney, small intestine, and testis. CD36 was higher expressed in lung, small intestine, colon, and pancreas. PRAT4A, the factor with the highest expression in spleen, revealed even higher expression levels in colon but not in any other solid organ. LL37 expression was higher in testis and GRP94 was higher in pancreas. Most other factors, revealed lower mRNA expression levels in solid organs as compared to spleen. Thus, the mRNA expression levels of most TLR accessory molecules are low in healthy solid organs, except for LBP, and CD36 in pancreas and lung, GRP94 in pancreas, LL37 in testis, and PRAT4A in colon.

### 2.2. TLR Accessory Molecule mRNA Expression in Adult Murine Tissues

Next we determined the mRNA expression levels of the same TLR accessory molecules in the same organs from 10 to 12 weeks old C57BL/6 mice. All molecules were constitutively expressed in mouse spleen but the mRNA levels of LBP, MD2, CD14, LL37, and TRIL were low (Figure 1B). In all other solid organs PRR mRNA levels were much lower as in spleen except for the following: LBP mRNA levels were higher in all organs except small intestine. CD36 mRNA levels were higher in lung, testis, and brain. CD14 and GRP94 mRNA levels were higher in lung and testis, CD14 in lung heart in pancreas, GRP94 in lung and GRN, and AEP mRNA levels were higher only in heart; PRAT4A in heart and pancreas, and AP3B1 in testis. Figure S1 compares the organ-specific TLR accessory molecule mRNA expression in humans and mice where white (human) and black (murine) bars indicate the x-fold induction *versus* respective spleen mRNA levels. The graph illustrates several discordant relative mRNA expressions between the two species. For example, human colon displayed higher relative mRNA levels of CD36, and PRAT4A as in mice. The relative mRNA levels of LBP were generally much higher in several murine organs, e.g., in lung, liver, kidney, and testis. CD14 was discordant especially in heart and pancreas. Thus, the mRNA expression levels of TLR accessory molecules differ in human and mouse organs.

### 2.3. TLR Accessory Molecule Expression upon Ischemia-Reperfusion Injury in Mice

**Figure 2:**
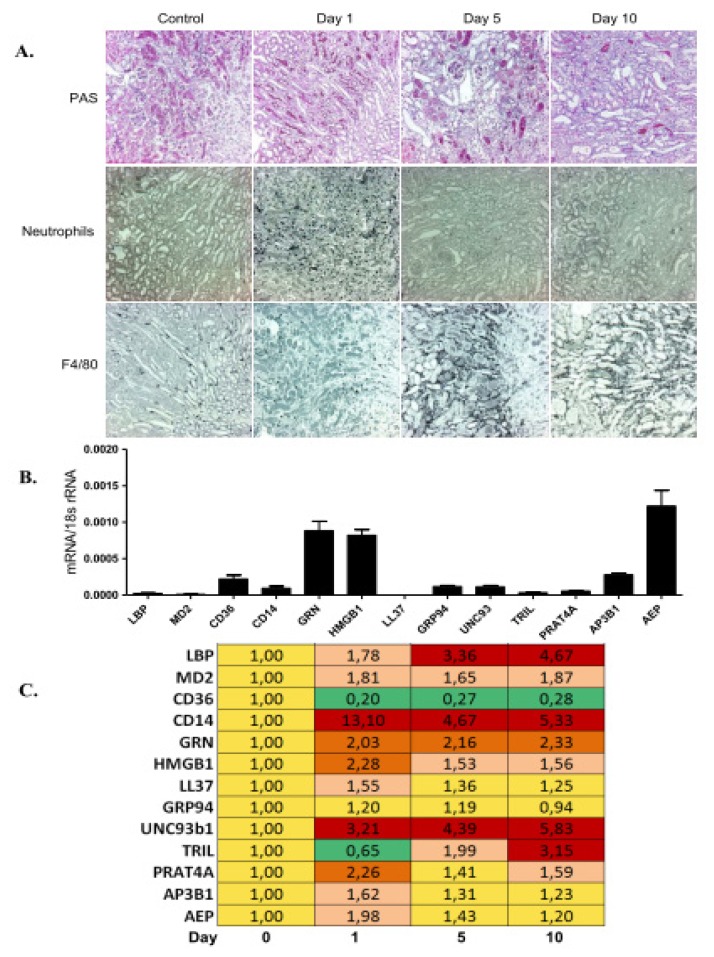
TLR accessory molecule mRNA expression during ischemia-reperfusion injury. (**A**) Renal ischemia-reperfusion injury was induced as described in methods. Representative images of renal sections stained with PAS or for neutrophils and macrophages are shown at three time points as indicated. Original magnification: ×200. Real-time PCR was performed on cDNAs derived from the kidney at baseline; and upon unilateral kidney ischemia reperfusion mice on day 1, 5 and 10; (**B**) The histogram represents the mRNA expression levels of different genes of the wild type kidney (control); (**C**) The table represents the relative expression of mRNA levels *versus* control using the colour code as illustrated.

As the constitutive expression levels of most TLR accessory molecules were low in most organs, we studied their induction in transient and progressive tissue inflammation. We selected ischemia-reperfusion injury upon renal pedicle clamping because this model is associated with a transient TLR2/4/MyD88-mediated sterile inflammation at day 1–2 in association with neutrophil infiltrates (Figure 2A) [23,24]. At this time point the mRNA expression levels of CD14 and most other TLR accessory molecules were induced as compared to baseline, except for CD36, TRIL, and GRP94 (Figure 2B,C). The subsequent resolution of inflammation from day 3–5 goes along with epithelial regeneration and lasts until day 10 in this model, which is associated with disappearance of neutrophils and an influx of alternatively-activated macrophages (Figure 2A) [25,26]. CD14 expression was also high during this phase but lower as during the injury phase (Figure 2C). Some molecule mRNA levels increased with time, such as LBP, UNC93b1, and TRIL. CD36 remained suppressed at any time point tested. TLR1, -4, -6, and -7 were strongly induced in post-ischemic kidneys at all time points, while TLR2, -3, and -9 were not much regulated *versus* controls (Figure S2A). Immunostaining for GRP94 displayed it to be expressed in a small subset of infiltrating CD45+ leukocytes; hence, its expression was not much affected by acute inflammation in the post-ischemic kidney (Figure S2B). In contrast, UNC93b1 was weakly expressed by vascular endothelial cells in the healthy kidney and, consistent with the mRNA expression profile, its staining intensity strongly increased in the post-ischemic kidney (Figure S2B). Together, in transient tissue inflammation the myeloid cell transmembrane molecule CD14 is up-regulated while CD36 is down-regulated.

### 2.4. TLR Accessory Molecule Expression in Systemic Autoimmunity of MRL/lpr Mice

**Figure 3:**
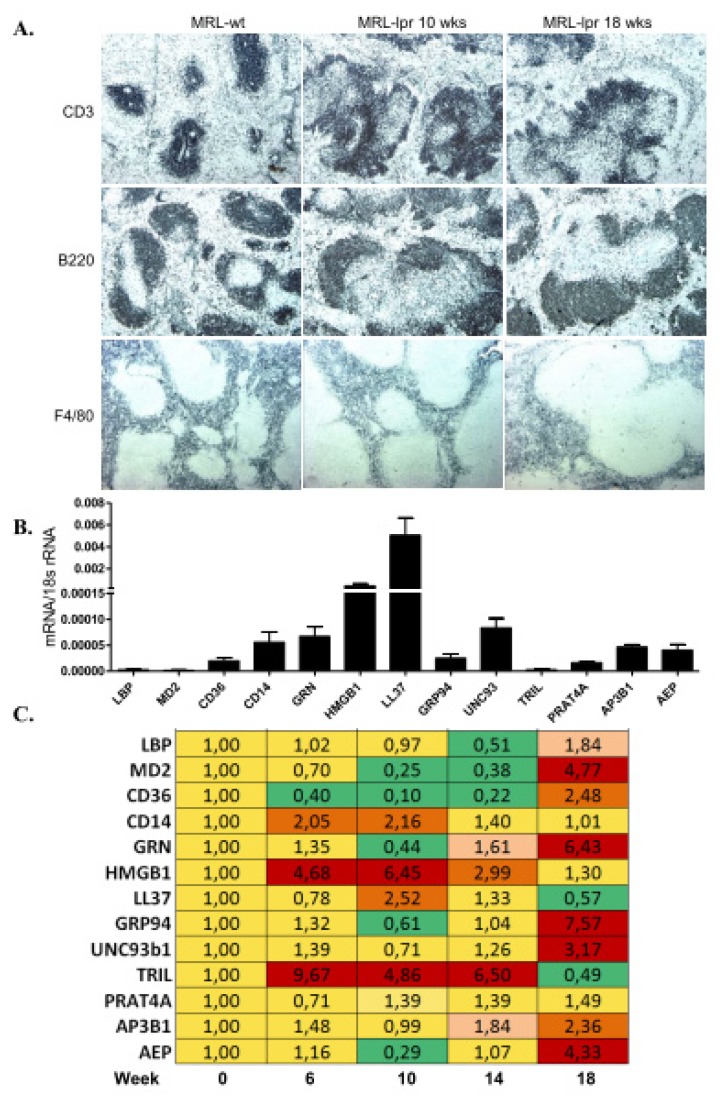
TLR accessory molecule mRNA expression in spleens of MRL-lpr mice. (**A**) MRL-lpr mice were used as a model of systemic autoimmunity. Spleen sections taken from 10 and 18 weeks old MRL-lpr mice were stained for CD3 T cells, B220 B cells and F4/80+ myeloid antigen-presenting cells. MRL wild type spleen represents control. Original magnification: ×100. Real-time PCR was performed on cDNAs derived from spleens of MRL-wild type controls on week 6; and of MRL-lpr mice at week 6, 10, 14 and 18; (**B**) The histogram represents the genes mRNA expression levels of different genes of the wild type spleen (control); (**C**) The table represents the relative expression of mRNA levels *versus* control using the colour code as illustrated.

Systemic autoimmunity is characterized by a lymphoproliferative syndrome with an expansion of various immune cell subsets in lymphoid organs and with tissue inflammation and progressive tissue remodelling in affected organs [27]. Especially, TLR7 and TLR9 have been implicated in the pathogenesis of SLE [2,3]. We selected the model of spontaneous SLE-like systemic autoimmunity of MRL/lpr mice to study the mRNA expression patterns of the TLR accessory molecules in spleens during the onset of autoimmunity. All expression profiles were referred to 10 weeks old MRL-wild type mice as baseline controls. MRL-lpr mouse spleens underwent a progressive increase in lymph follicle size and an expansion of germinal centres (Figure 3A). All TLR accessory molecules were expressed at baseline, of which CD14, HMGB1, and TRIL were induced at 6, 10, and 14 weeks in MRL-lpr mice (Figure 3B,C). In contrast, AEP, AP3B1, GRP94, UNC93b1, MD2, and GFN were not induced before 18 weeks of age. TLR mRNA expression was not much regulated except for a 3,6 fold induction of TLR4 at week 10 (Figure S3A). Immunostaining for GRP94 displayed positivity in the T cell zone of the follicles close to the central arteriole and positivity increased with the expansion of this zone at 18 weeks of age (Figure S3B). UNC93b1 protein did not seem to be expressed in lymphocytes but mostly in the endothelium of the perifollicular sinus (Figure S3B). Thus, some of the molecules that are involved in TLR7 and TLR9 activation, such as HMGB1, GRP94, UNC93b1, AP3B1, and AEP are induced in spleen during the initiation and progression of SLE.

### 2.5. TLR Accessory Molecule Expression in Progressive Lupus Nephritis of MRL/lpr Mice

**Figure 4:**
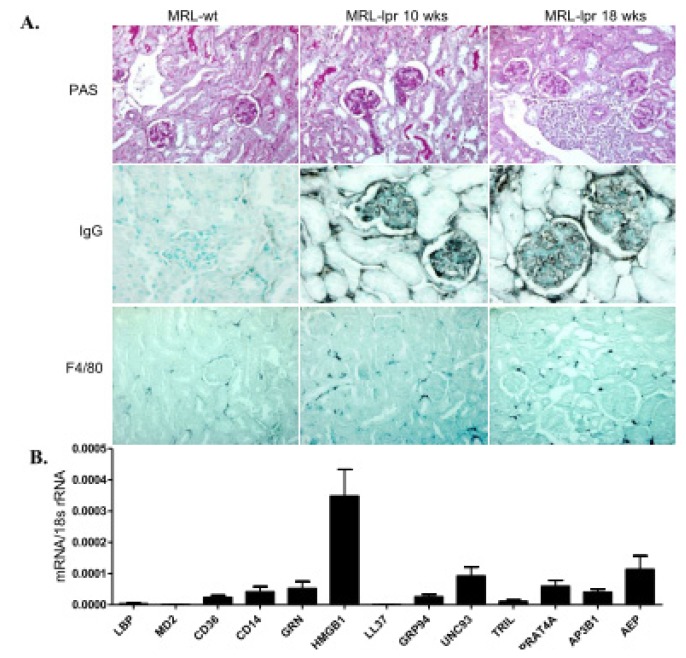

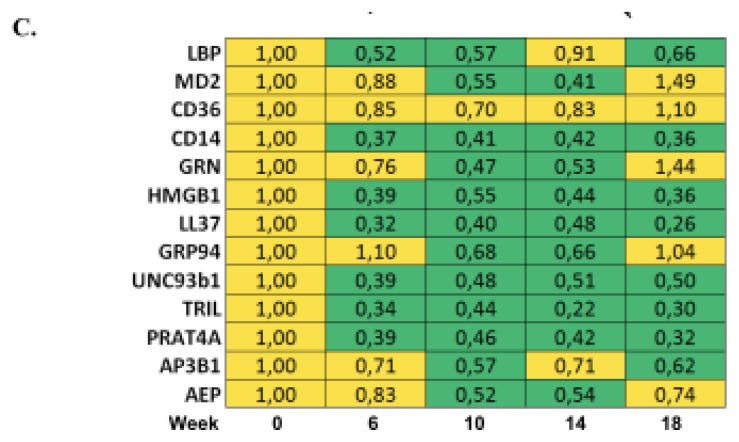
TLR accessory molecule mRNA expression in the kidney of MRL-lpr mice. (**A**) Kidney sections taken from 10 and 18 weeks old MRL-lpr mice were stained with PAS and for IgG and F4/80+ myeloid antigen-presenting cells. MRL wild type kidney represents control. Original magnification: ×200. Real-time PCR was performed on cDNAs derived from kidneys of MRL-WT controls on week 6; and of MRL-lpr mice at week 6, 10, 14 and 18; (**B**) The histogram represents the genes mRNA expression levels of different genes of the wild type spleen (control); (**C**) The table represents the relative expression of mRNA levels *versus* control using the colour code as illustrated.

The organ manifestations in SLE develop as a consequence of SLE-dependent persistent immune complex disease and the infiltration of autoantigen-specific T and B cells (Figure 4A) [28,29]. Thus, the pathogenesis of lupus nephritis and its progressive tissue remodelling can be separated into extrarenal and intrarenal pathomechanisms [28]. Therefore, we assessed the TLR accessory molecule mRNA expression profiles also in kidneys during the progression of lupus nephritis. Kidneys of 10 weeks old MRL-wild type mice expressed all of these factors and were used as baseline controls (Figure 4B,C). However, the renal mRNA expression levels of all TLR accessory co-factors were down-regulated during the progression of lupus nephritis. Along this, TLR1, -6, and -7 were induced in lupus nephritis at week 18, while TLR2, -3, -4, and -9 were not much regulated *versus* baseline (Figure S4A). However, it is of note that TLR2 and TLR9 were already expressed at much higher levels under baseline conditions. Immunostaining displayed GRP94 to be expressed in a small subset of the infiltrating CD45+ leukocytes, while UNC93b1 stained parietal epithelial cells along Bowman’s capsule and vascular endothelial cells in a similar manner at early and late time points (Figure S4B). Thus, tissue inflammation and remodelling during progressive lupus nephritis is not associated with a similar induction of TLR accessory molecules as observed in spleen.

### 2.6. TLR Accessory Molecule Expression in Progressive Tissue Fibrosis

**Figure 5:**
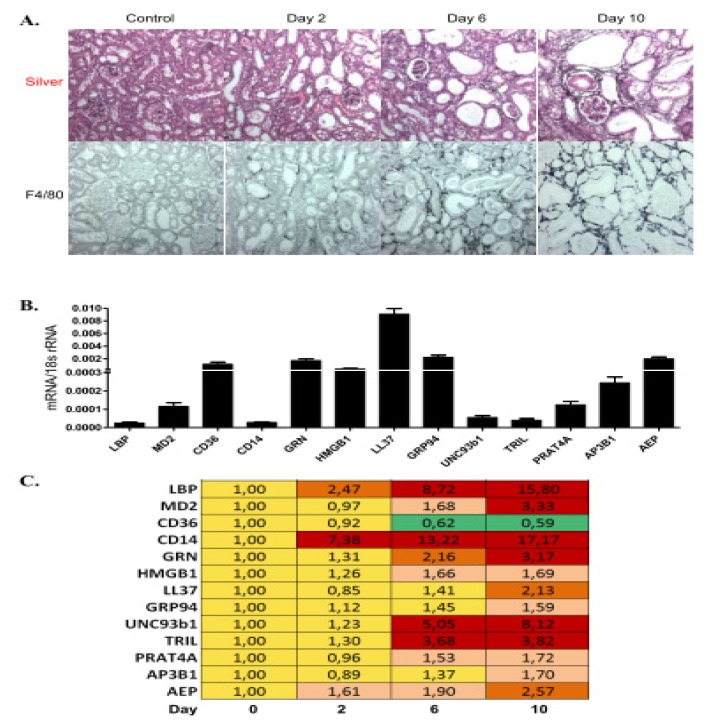
TLR accessory molecule mRNA expression upon unilateral ureteral ligation. (**A**) Kidney sections were taken at day 2, 6, and 10 after unilateral ureteral obstruction (UUO) and were stained with silver and F4/80+ myeloid antigen-presenting cells. A contralateral kidney represents control. Original magnification: ×200. Real-time PCR was performed on cDNAs derived from baseline control kidneys of C57BL/6 mice; or of kidneys upon unilateral ureteral ligation (UUO) at day 2, 6, and 10; (**B**) The histogram represents the genes mRNA expression levels of different genes at baseline (control); (**C**) The table represents the relative expression of mRNA levels *versus* control using the colour code as illustrated.

Chronic tissue remodelling is often associated with progressive and irreversible tissue fibrosis. Immune cells contribute to this process by maintaining inflammation and by producing pro-fibrotic cytokines [30,31]. We used the model of unilateral ureter obstruction, which leads to rapid kidney atrophy in association with a progressive macrophage infiltrates and interstitial fibrosis (Figure 5A) [32]. We assessed the TLR accessory molecule mRNA expression profiles at three time points in obstructed kidneys. Two days after ureter ligation only LBP and CD14 were induced compared to baseline and remained the most strongly induced genes also at the later time points (Figure 5B,C). Up to day 10 all other genes were also progressively up-regulated, with the exception that only CD36 was down-regulated (Figure 5B). Along this, TLR1, -2, -4, -6, and -7 were strongly induced upon ureteral obstruction with time, while TLR3 and -9 were not much regulated *versus* baseline (Figure S5A). Immunostaining displayed GRP94 to be expressed in a small subset of the infiltrating CD45+ leukocytes, while UNC93b1 stained parietal epithelial cells along Bowman’s capsule and vascular endothelial cells. Consistent with the respective mRNA expression profiles, only UNC93b1 positivity strongly increased from day 2 to day 10 of obstruction and *versus* non-obstructed control kidneys (Figure S5B). Thus, progressive tissue fibrosis is associated with a broad induction of TLR accessory molecules, except CD36.

TLR signalling contributes to acute and chronic forms of tissue inflammation, though in different ways [33]. For example, TLR9 signalling promotes antiviral host defence [1] as well as tissue inflammation during the progression of autoimmune tissue injury, but it suppresses the development of systemic autoimmunity by inhibiting TLR7 signalling in lymphoid organs [2,34]. As another example, TLRs contribute to tissue fibrosis by promoting M2 macrophage polarization [35], probably by inducing immunoregulatory factors such as IRAK-M and IRF4 [36,37]. As TLRs are also differentially expressed in human and mouse immune cell subsets [38], we characterized the respective species- and disease-specific expression patterns of TLR accessory molecules. Our selection of TLR co-factors encompassed soluble mediators, transmembrane molecules, as well as intracellular factors that regulate the trafficking and activation of TLRs [4].

One finding of our study is that there are significant differences in the relative mRNA expression profiles of the TLR accessory molecules in mice and humans. This does apply in a similar manner to the pattern recognition receptors themselves such as the TLRs [38], the NLRs, RLH, and inflammasomes [39] as well as the C-type lectin receptors [40]. Thus, species-specific expression patterns need to be considered in the interpretation of either data and human studies need to verify the functional roles of single co-factors suggested by studies performed in rodents.

Ischemia-reperfusion injury is a transient form of sterile inflammation, characterized by a serial influx of neutrophils and pro-inflammatory macrophages that contribute to sterile inflammation followed by anti-inflammatory macrophages that contribute to tissue regeneration [25,41–43]. The early induction of CD14 was probably related to the neutrophil response while the induction of UNC93b1 remains unclear in this context. TLR9 was not much induced during the disease but TLR9 expression was already high from baseline. CD36 was the only factor that was persistently down-regulated, similar to what we had found in the progressive UUO model. The healing phase also induced LBP and TRIL, but a causal role for this process remains speculative. There is first evidence that TLR2 and TLR4 drive epithelial regeneration upon kidney injury but the role of the accessory molecules remain to be defined [44–46].

The pathogenesis of lupus nephritis is driven by immune deregulation in lymphoid tissues, where the loss of tolerance against nuclear autoantigens leads to a TLR7 and-9-dependent activation of antigen-presenting cells that drive the expansion of autoreactive lymphocytes [2,28]. This may explain why we hardly found any regulation in the TLR accessory molecules in the kidney but rather in spleens of MRL-lpr mice. Especially HMGB1 and TRIL were induced in spleens during early SLE, while accessory molecules to TLR7 and TLR9 (UNC93b1, GRP94, GRN, AP3B1, AEP) were induced only at later time points. The specific contribution of these molecules in SLE has not yet been studied in detail, but it seems likely that they act as co-factors for the TLR7 and -9 dependent disease activity of SLE [2,47,48].

Tissue fibrosis was studied using the model of UUO, which leads to renal interstitial fibrosis and tubular atrophy within a few days upon surgery [32]. Most TLR accessory molecules were induced along the progressive nature of the disease, except for CD36. The reason for this selective down regulation is unclear, but the progressive induction of all other TLR accessory parallels the progressive increase of mononuclear phagocyte accumulation in the fibrotic kidney. T cell infiltrates accompany these infiltrates but most of these molecules are rather expressed by myeloid cells [4]. LBP and CD14 were most strongly induced suggesting a role for TLR4 in this model. In fact, two studies have documented an attenuated renal fibrosis and M2 macrophage recruitment in Tlr4-deficient mice with UUO [35,49], while other studies do not support an involvement of the TLR4/Myd88 signalling pathway [50,51].

## 3. Experimental Section

### 3.1. Human Solid Organ cDNA Preparation

Human solid organ pre-normalized cDNAs derived from poly-(A)-selected DNase-treated RNAs purified from pools of healthy human tissues were obtained from Clontech, Mountain View, CA, USA. An equal amount of cDNA from each individual preparation was used as a template in PCR with primers for each of the four tested reference genes (α-tubulin, β-actin, GAPDH/G3PDH, phospholipase A2). An 18 s ribosomal unit was not detectable in poly-(A)-purified RNAs. The PCR product band was determined by video imaging and computer analysis, and band intensity was determined. If necessary, the concentration of individual cDNA preparations was then adjusted so that the average band intensity for the reference genes used to normalize the panel varied no more than 20%. As only a single pool was available for each organ no studies on biological replicates allowing statistics could be performed. According to Clontech all human samples were purchased and imported in accordance with all local laws and regulations. Donors were tested to be negative for HIV, hepatitis B virus, and hepatitis C virus. Further exclusion criteria were as follows: manifest infections during the last 4 weeks, fever, symptomatic allergies, abnormal blood cell counts, increased liver enzymes, or medication of any kind except vitamins and oral contraceptives. The study was approved by the Ethics committee of Klinikum der Universität München.

### 3.2. Animal Models of Transient and Progressive Tissue Inflammation

Groups of eight week old sex-matched C57BL/6 mice (*n* = 5–10) underwent unilateral renal pedicle clamping as a model of ischemia-reperfusion as described [52]. In brief, mice were anesthetized before the left renal pedicle was clamped for 45 min with a microaneurysm clamp via 1 cm flank incisions (Medicon, Tuttlingen, Germany). Body temperature was continuously measured with a rectal probe and maintained at 36–37 °C throughout the procedure by placing the mice on a heating pad. After clamp removal the kidney was inspected for restoration of blood flow as evidenced by returning to its original colour before closing the wound with standard sutures. To maintain fluid balance, all mice were supplemented with 0.5 mL of saline. Mice were sacrificed 1, 5, and 10 days after surgery and pieces from IRI and contralateral (sham) kidneys were either snap frozen in liquid nitrogen or fixed in 10% buffered formalin. For the lupus model, groups of female MRL/Wt or MRL/lpr mice with spontaneous lupus-like autoimmunity were sacrificed at age of 6, 10, 14, and 18 weeks as described [53]. UUO was performed in 6–8 weeks old, sex-matched C57BL/6 mice mice as previously described [50]. In brief, under general ether anesthesia, unilateral ureteral ligation resulting in UUO was performed by ligating the left distal ureter with a 2/0 Mersilene suture through a low midline supravesical abdominal incision of 1 cm. Unobstructed contralateral kidneys served as controls. Groups of mice were killed at 2, 6, and 10 day after UUO by cervical dislocation under general anesthesia with inhaled ether. Kidneys and spleens were harvested for RNA isolation and RT-PCR [54]. All experimental procedures were performed according to the German animal care and ethics legislation and had been approved by the local government authorities.

### 3.3. Mouse Solid Organ cDNA Preparation for qRT-PCR Experiments

Ten to twelve week old adult C57BL/6 mice were purchased from Charles River, Sulzfeld, Germany and maintained under standard conditions and 12 h light/dark cycle. Animals were housed in polypropylene cages and allowed free access to food and water *ad libitum*. Mice were sacrificed by cervical dislocation and high quality, DNA-free RNA was isolated from freshly harvested tissues as described [39]. Tissues were kept in RNAlater reagent and RNA was isolated from same tissue mass (10 mg) with Pure Link RNA Mini Kit according to manufacture instructions (sample size normalization). Samples were digested with DNAse solution and additional washing steps were performed to remove traces of DNAse. Concentrations of aqueous RNA samples were measured with NanoDrop 1000 Spectrophotometer. Only samples with absorbance 260/280 between 1.95 and 2.05 were considered as pure RNA, the integrity of the total RNA was determined by electrophoresis on 2% (*w*/*v*) agarose gels as described.

### 3.4. Quantitative Real-Time RT-PCR

Good quality 1 μg of RNA was proceeded to cDNA (second normalization step) using thermo stable RNAse inhibitor during reverse transcription as described [40]. Reverse transcriptions were performed with the same reaction mix containing Superscript II reverse transcriptase (Invitrogen, NY, USA), dNTPs, hexanucleotides, linear acrylamid, DTT and 5× Superscript buffers using standard protocol. Same amounts of RNA (1 μg) was heated to 65 °C for 5 min, and then put on ice. RT-PCR reaction was performed at 42 °C for 90 min. Mouse cDNAs from organs and disease models were additionally normalized with α-tubulin, β-actin, GAPDH/G3PDH, phospholipase A2, β-2-microglobulin and ribosomal unit 18 s. GAPDH was chosen for analysis of the human and mouse organs and ribosomal protein 18 s was chosen as a reference gene for disease models based on its low variation in disease models (Tables S1–S3). Geometric mean, arithmetic mean, minimal value, maximal value, standard deviation, variance and coefficient of variance were calculated. GAPDH and 18 s were chosen as the most stably expressed in organs and disease models, respectively. TLR accessory molecule mRNAs expression in human and mouse solid organs cDNA was quantified by real-time RT-PCR using GAPDH as housekeeping genes. Each PCR reaction (20 μL) contained 10× *Taq* Polymerase Buffer, *Taq* Polymerase, dNTPs, BSA, PCR Optimizer, SYBR green solution, MgCl2, gene specific primers and 0.2 μL of synthesized cDNA. SYBR Green Dye detection system (SYBR Green I 96 protocol LC480 Roche running program) was used for amplification. Quantitative real-time PCR was performed on Light Cycler 480 (Roche, Mannheim, Germany). Each amplification step included initiation phase 95 °C, annealing phase 60 °C and amplification phase 72 °C and was repeated 45 times. Gene-specific primers (300 nM, Metabion, Martinsried, Germany) were used as listed in Tables 1 and S4. Controls consisting of ddH2O were negative for target and housekeeper genes. Primers were designed to be cDNA specific and to target possibly all known transcripts of genes of interest. *In silico* specificity screen (BLAST) was performed. The lengths of amplicons were between 80 and 130 bp. The kinetics of the PCR amplification (efficiency) was calculated for every set of primers. The efficiency-corrected quantification was performed automatically by the LightCycler 480 based on external standard curves describing the PCR efficiencies of the target and the reference gene: [ratio = EtargetΔCPtarget (control – sample)/ErefΔCPref (control – sample)]. To reduce the risk of false positive Cp the high confidence algorithm was used. All the samples that during the amplification reaction did not rise above the background fluorescence (crossing point Cp or quantification cycle Cq) of 40 cycles were described as not detected (n.d. = not detected in the figures). Crossing points between 5 and 40 cycles were considered as detectable. The melting curves profiles were analyzed for every sample to detect unspecific products and primer dimers. Products were visualized on agarose gels, extracted and analyzed for sequence.

### 3.5. Histopathology

Kidney tissues were fixed in 4% neutral-buffered formalin, dehydrated in graded alcohols and embedded in paraffin. For periodic acid-Schiff (PAS) staining or immunostaining, 4 μm sections were deparaffinised, rehydrated, transferred into citrate buffer, and either autoclaved or microwave treated for antigen retrieval and processed as described [26]. The following primary antibodies were used: anti-CD3+, anti-F4/80 (both Serotec, Oxford, UK), mIgG (Caltag Laboratories, Burlingame, CA, USA), anti-B220 (BioLegend, San Diego, CA, USA), anti-Ly-6G (BD Pharmingen, Heidelberg, Germany).

## 4. Conclusions

In summary, we identified significant differences in the mRNA expression of TLR accessory molecules in human and mouse solid organs and in their regulation during transient and progressive tissue inflammation. These findings can help to generate novel hypotheses on the role of single TLR accessory molecules in selected organ pathologies. Furthermore, the species-specific expression of single TLR co-factors need to be considered in the interpretation of either data, and human studies need to verify the functional roles of single co-factors suggested by studies performed in rodents.

## Supplementary Information



## Figures and Tables

**Table 1:** Primers used for qPCR and its efficiency.

Heading	Right primer sequence	Left primer sequence	Accession Nr.	Efficiency
Human				

LBP	CCGATTTCTGGATCATTTCG	GTGGACATGTCGGGAGACTT	NM_004139	2.42
MD2	TCCCTTGAAGGAGAATGATATTG	AATCTTCCAAAGCGCAAAGA	NM_015364	2.17
CD36	TCAATTCGTCTAATCATTGGAAA	GCAAGACTCTGGAGCCAGTC	NM_001001547	2.14
CD14	CTCACAAGGTTCTGGCGTG	TGAGCTCAGAGGTTCGGAAG	NM_000591	1.80
GRN	CCCTGAGACGGTAAAGATGC	CGTCCCCTTCTGGACAAAT	NM_002087	1.80
HMGB1	AGGATCTCCTTTGCCCATGT	TGAGCTCCATAGAGACAGCG	NM_002128	1.96
LL37	GTGACTGCTGTGTCGTCCTG	GCTAACCTCTACCGCCTCCT	NM_004345	1.97
GRP94	TCCAATTCAAGGTAATCAGATGC	TGTAATTGCTGACCCAAGAGG	NM_003299	1.73
UNC93b1	GCGAGGAACATCATCCACTT	GATGGGCATCAACGTGACT	NM_030930	2.14
TRIL	ACCGCCTCCACCGTCAGGTT	CACGGAGCACCAGGAGCGTG	NM_014817	2.08
PRAT4A	AGGTCTTCCTCCTGGTGGTT	AGAGGTGGCTGACCTCAAGA	NM_006586	2.51
AP3B1	TCGTTGGTACAAATGCAGGA	CTTCCCACACCAGCTCTTTC	NM_003664	2.15
AEP	AACCATTCTGCACCTTGGAG	CGCGAGTTCTCACGGTC	NM_005606	1.93

Mouse				

LBP	GGAGGTCCACTGAAATGGTG	TCGCCATCTCTGACTCTTCC	NM_008489	1,94
MD2	GGCACAGAACTTCCTTACGC	TGCATGTTGAGTTCATTCCAA	NM_016923	1.76
CD36	CCTGCAAATGTCAGAGGAAA	GCGACATGATTAATGGCACA	NM_007643	1.91
CD14	CGCAGGAAAAGTTGAGCGAGTG	TTGAACCTCCGCAACGTGTCGT	NM_009841	2.12
GRN	GGTGGCAGAGTCAGGACATT	GGTGTGTCTTGTGGTGATGG	NM_008175	1.94
HMGB1	AGGATCTCCTTTGCCCATGT	TGAGCTCCATAGAGACAGCG	NM_010439	1.93
LL37	GCCACATACAGTCTCCTTCACTC	CTTCAACCAGCAGTCCCTAGAC	NM_009921	2.44
GRP94	TTGTGTCCAATTCAAGGTAATCA	TTGCTGACCCAAGAGGAAAC	NM_011631	1.81
UNC93b1	GCTATGAGCAGGTATGCCAGTC	CTACAGTGGCTTTGAGGTGCTC	NM_019449	2.10
TRIL	TTGTTCCCCAGGTACAGCTC	CTTCATCACCAACATCACCG	NM_025817	1.99
PRAT4A	CTGTACCAGTCCTCGATCACCT	GTCAAGGTGGTGATGGACATCC	NM_028065	2.45
AP3B1	TGGCAGAATCTTTGTTGCTCT	ACCTCGACCATCTCTCCCTC	NM_009680	2.57
AEP	GAGAAGCACAGCCACTCTCC	TCCCACGGTTCTGCAGTC	NM_011175	2.01
