# Supplementary material for: Toll-Like Receptor and Accessory Molecule mRNA Expression in Humans and Mice as Well as in Murine Autoimmunity, Transient Inflammation, and Progressive Fibrosis
Source: Int J Mol Sci. 2013 Jun 26;14(7):13213–30. doi: 10.3390/ijms140713213 (PMC3742183; doi:10.3390/ijms140713213)
Supplement: Supplementary file 1 [file ijms-14-13213-s001.pdf]

# Supplementary Information

**Figure S1.** The relative expression of TLR accessory molecular mRNA expression in different organs compared to the spleen. The respective relative murine (black bars) and human (open bars) TLR accessory molecular mRNA levels from Figure 1A,B are illustrated. The *x*-axis marks a ratio of 1, hence, positive values indicate stronger expression in humans. Negative values indicate stronger expression in mice. The *y*-axis marks the fold-change in each direction. Note that the scale of the *y*-axis is different for each organ. Data represent means ± SEM.

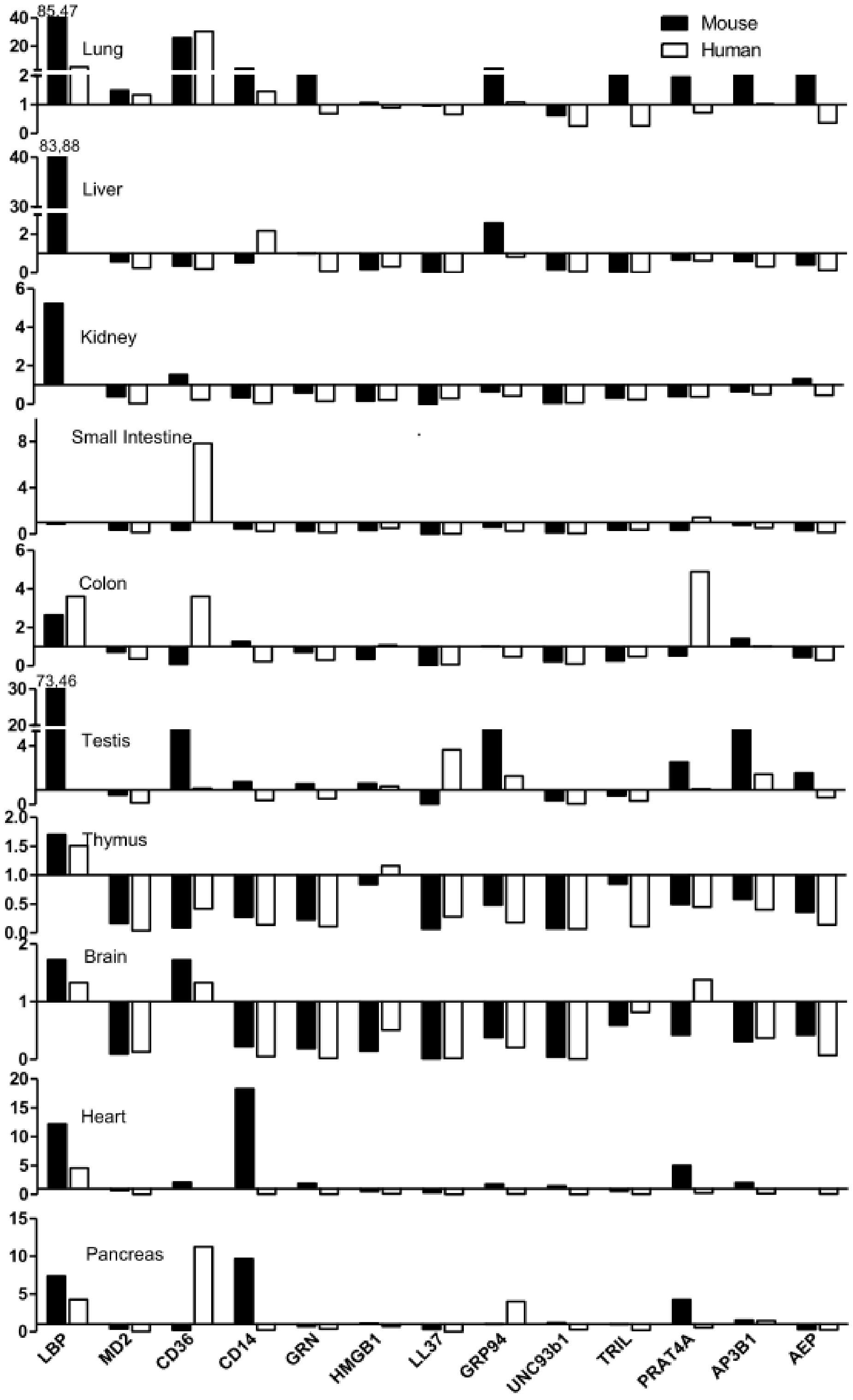

**Figure S2.** (**A**) Toll like receptors mRNA expression during ischemia-reperfusion injury. The histogram in A represents the genes mRNA expression levels of different genes of the wild type kidney (control). The table in A represents the relative expression of mRNA levels versus control using the colour code as illustrated; (**B**) Immunofluorescence staining of CD45 (green), GRP94 (red) and UNC93b1 (red) in kidney of ischemia reperfusion injury.

**A.**

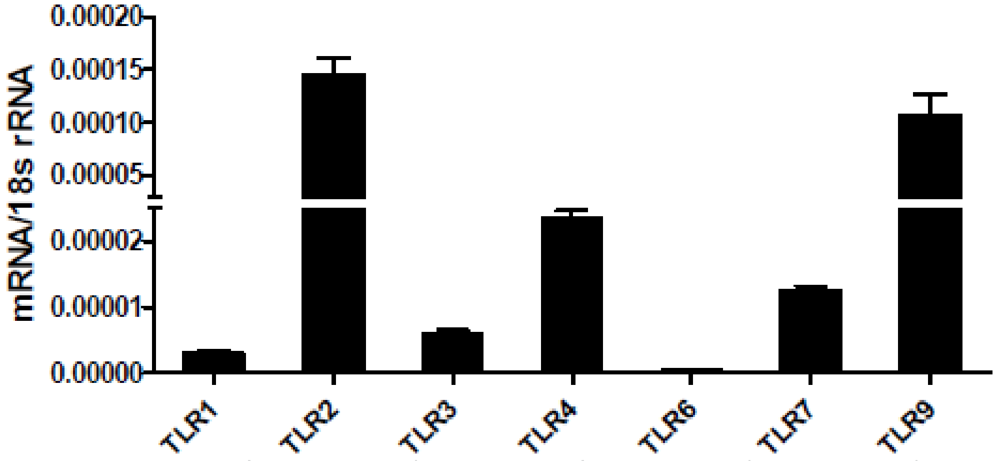


| | | | | |
|---|---|---|---|---|
| TLR1 | 1,00 | 7,87 | 8,60 | 9,55 |
| TLR2 | 1,00 | 1,77 | 1,49 | 1,45 |
| TLR3 | 1,00 | 1,82 | 1,52 | 1,57 |
| TLR4 | 1,00 | 4,53 | 3,05 | 3,50 |
| TLR6 | 1,00 | 13,11 | 9,13 | 4,94 |
| TLR7 | 1,00 | 4,68 | 5,98 | 6,15 |
| TLR9 | 1,00 | 2,31 | 1,19 | 1,55 |
| Day | 0 | 1 | 5 | 10 |

**B.**

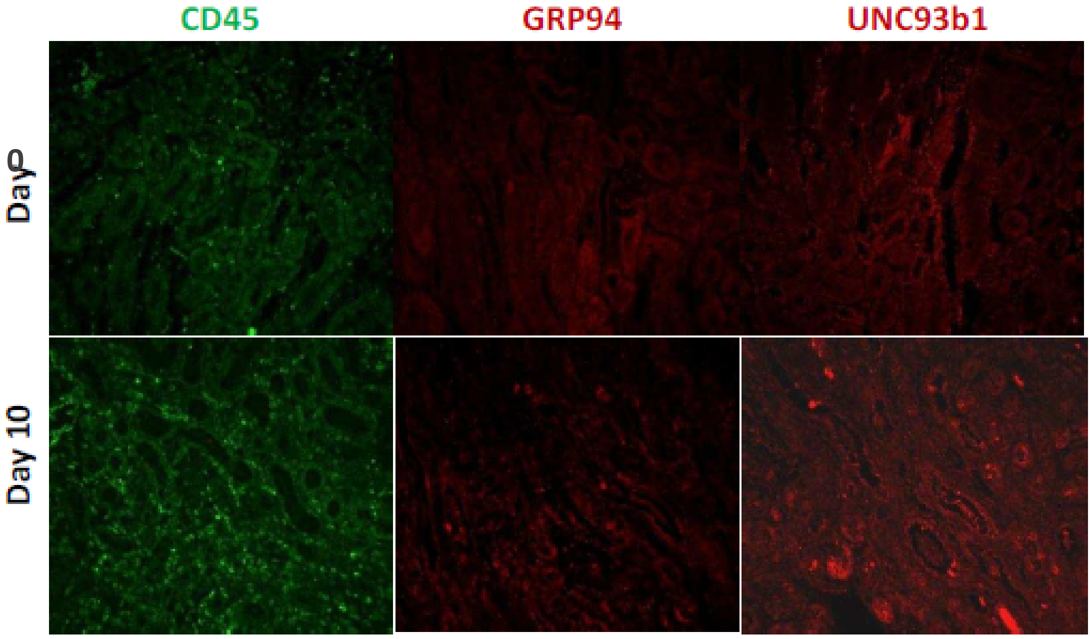

**Figure S3.** (**A**) Toll like receptors mRNA expression in spleens of MRL-lpr mice. The histogram in A represents the genes mRNA expression levels of different genes of the wild type spleen (control). The table in A represents the relative expression of mRNA levels versus control using the colour code as illustrated; (**B**) Immunofluorescence staining of CD45 (green), GRP94 (red) and UNC93b1 (red) in spleens of MRL-lpr mice.

A.

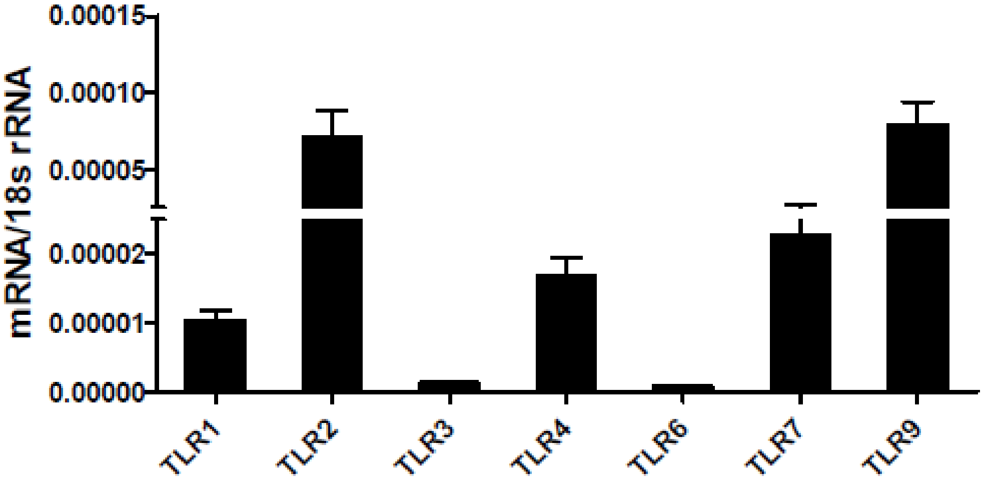


| | | | | | |
|---|---|---|---|---|---|
| TLR1 | 1,00 | 0,96 | 0,47 | 0,56 | 2,44 |
| TLR2 | 1,00 | 0,82 | 2,39 | 0,52 | 0,38 |
| TLR3 | 1,00 | 1,05 | 1,78 | 0,87 | 1,25 |
| TLR4 | 1,00 | 2,66 | 3,61 | 1,20 | 1,12 |
| TLR6 | 1,00 | 1,25 | 0,74 | 0,62 | 1,70 |
| TLR7 | 1,00 | 0,62 | 0,66 | 0,59 | 2,02 |
| TLR9 | 1,00 | 0,69 | 1,78 | 2,16 | 0,91 |
| Week | 0 | 6 | 10 | 14 | 18 |

B.

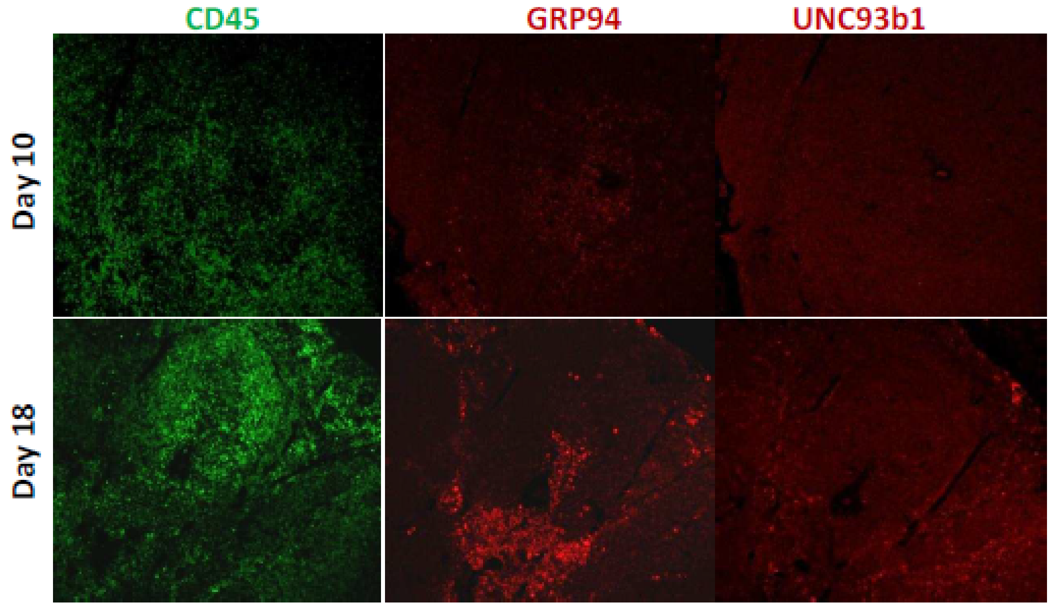

**Figure S4.** Toll like receptors mRNA expression in kidney of MRL-lpr mice. The histogram in A represents the genes mRNA expression levels of different genes of the wild type spleen (control). The table in (**A**) represents the relative expression of mRNA levels versus control using the colour code as illustrated (**B**). Immunofluorescence staining of CD45 (green), GRP94 (red) and UNC93b1 (red) in kidney of MRL-lpr mice.

A.

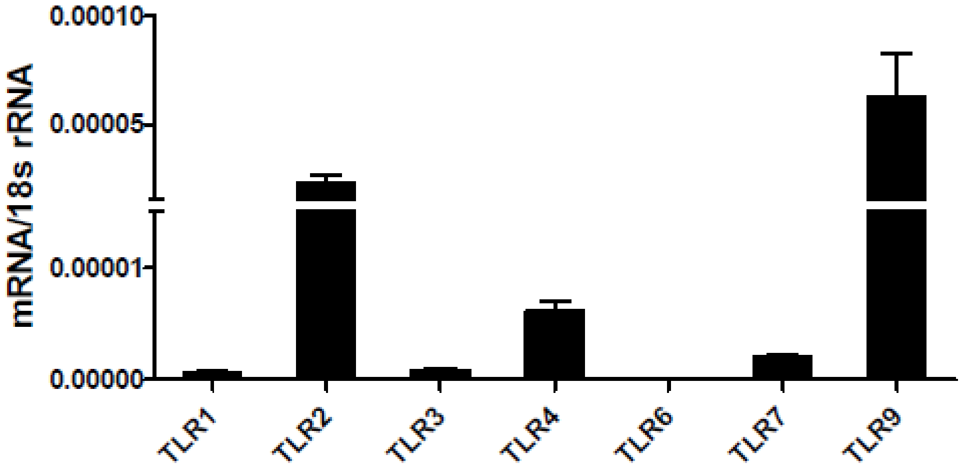


| | | | | | |
|---|---|---|---|---|---|
| TLR1 | 1,00 | 1,94 | 0,79 | 1,58 | 4,03 |
| TLR2 | 1,00 | 0,94 | 0,84 | 0,83 | 0,33 |
| TLR3 | 1,00 | 1,04 | 0,89 | 0,84 | 1,29 |
| TLR4 | 1,00 | 0,83 | 0,78 | 0,70 | 1,02 |
| TLR6 | 1,00 | 0,88 | 0,93 | 0,67 | 2,58 |
| TLR7 | 1,00 | 0,94 | 0,71 | 0,98 | 2,62 |
| TLR9 | 1,00 | 0,90 | 0,40 | 0,60 | 0,82 |
| Week | 0 | 6 | 10 | 14 | 18 |

B.

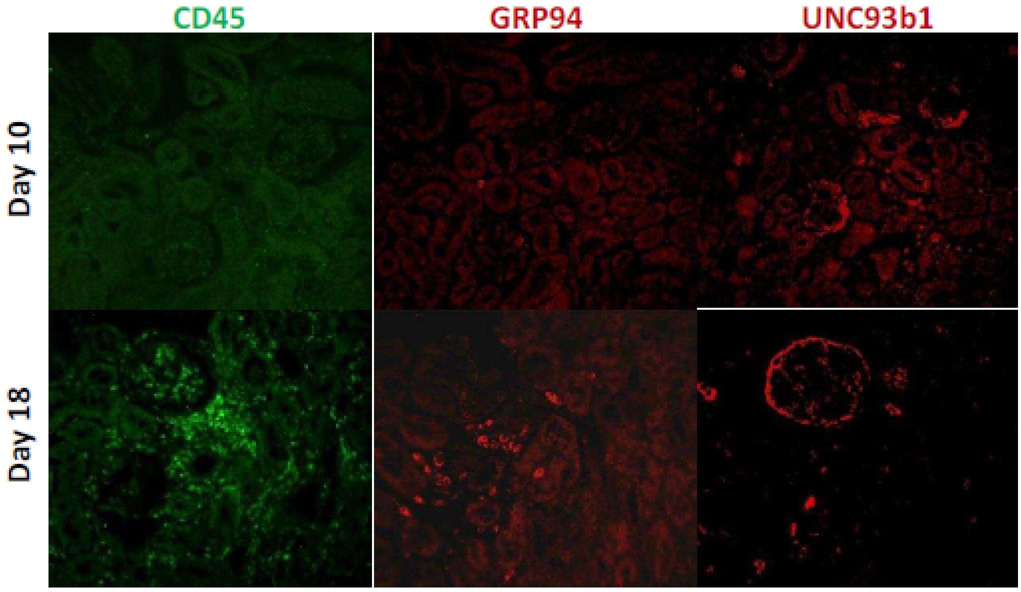

**Figure S5.** Toll like receptors mRNA expression upon unilateral ureteral ligation. The histogram in A represents the genes mRNA expression levels of different genes of the wild type spleen (control). The table in (**A**) represents the relative expression of mRNA levels versus control using the colour code as illustrated (**B**). Immunofluorescence staining of CD45 (green), GRP94 (red) and UNC93b1 (red) in kidney of unilateral ureteral ligation.

A

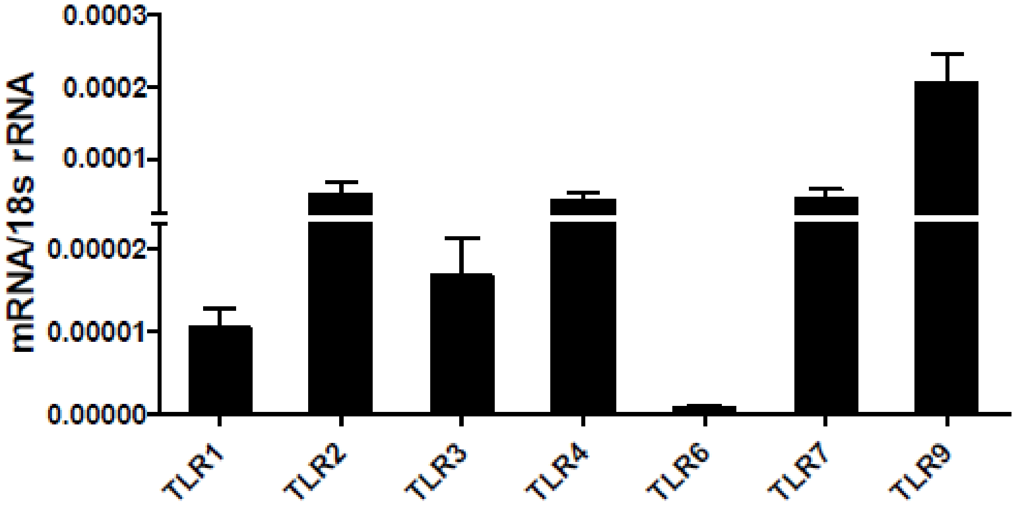


| | | | | |
|---|---|---|---|---|
| TLR1 | 1,00 | 0,90 | 4,17 | 5,56 |
| TLR2 | 1,00 | 2,38 | 5,37 | 5,42 |
| TLR3 | 1,00 | 0,66 | 1,20 | 1,56 |
| TLR4 | 1,00 | 0,80 | 2,20 | 3,28 |
| TLR6 | 1,00 | 0,78 | 3,31 | 6,76 |
| TLR7 | 1,00 | 0,69 | 2,64 | 3,89 |
| TLR9 | 1,00 | 0,62 | 1,32 | 1,71 |
| Day | 0 | 2 | 6 | 10 |

B

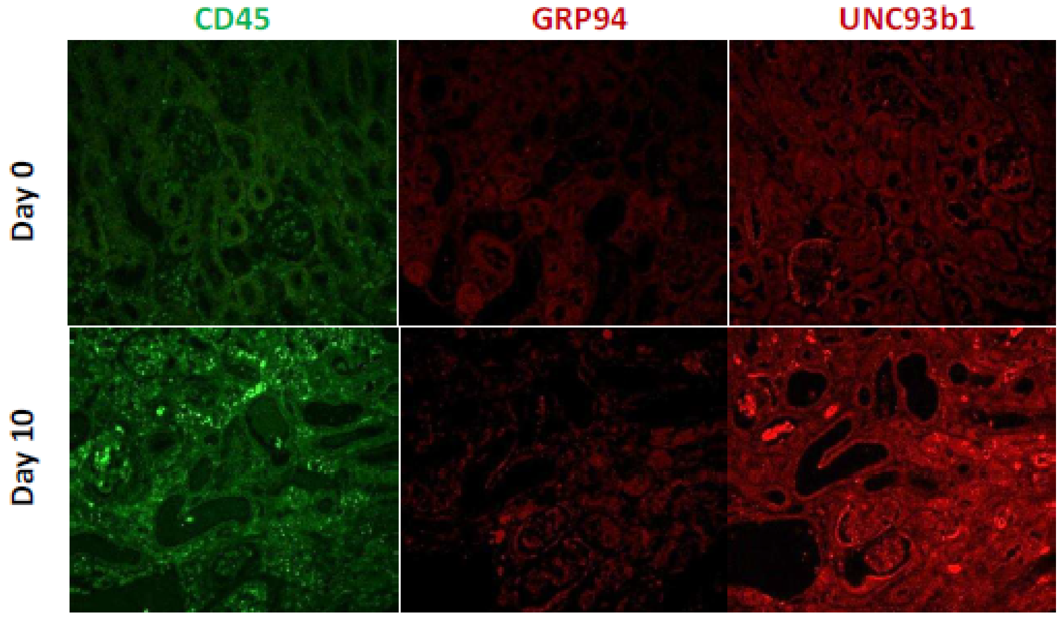

**Table S1.** Crossing point values of mouse housekeeping genes.

| | **18 s** | **GAPDH** |
|---|---|---|
| Number of samples | 11 | 11 |
| Geometric mean | 8.85 | 17.24 |
| Arithmetic mean | 8.86 | 17.26 |
| Minimum | 8.50 | 16.03 |
| Maximum | 9.21 | 18.21 |
| Standard deviation | 0.22 | 0.81 |
| Variance | 0.05 | 0.66 |
| Coefficient of variance (%) | 0.005 | 0.11 |

**Table S2.** Crossing point values of human GAPDH.

| | **GAPDH** |
|---|---|
| Number of samples | 11 |
| Geometric mean | 24.40 |
| Arithmetic mean | 24.44 |
| Minimum | 22.53 |
| Maximum | 26.90 |
| Standard deviation | 1.38 |
| Variance | 1.91 |
| Coefficient of variance (%) | 0.46 |

**Table S3.** Crossing point values of mouse 18s in disease models.

| | **IRI** | **Lupus spleen** | **Lupus kidney** | **UUO** |
|---|---|---|---|---|
| Number of samples | 23 | 23 | 43 | 21 |
| Geometric mean | 10.39 | 9.55 | 10.06 | 9.48 |
| Arithmetic mean | 10.40 | 9.65 | 10.09 | 9.52 |
| Minimum | 9.75 | 7.72 | 8.55 | 8.69 |
| Maximum | 11.23 | 11.17 | 12.09 | 12.30 |
| Standard deviation | 0.42 | 1.42 | 0.85 | 0.88 |
| Variance | 0.18 | 2.01 | 0.73 | 0.78 |
| Coefficient of variance (%) | 0.01 | 0.19 | 0.07 | 0.07 |

IRI, ischemia-reperfusion injury; UUO, unilateral ureteral obstruction.

**Table S4.** TLR Primers used for qPCR.

| | **Right primer sequence** | **Left primer sequence** | **Accession Nr.** |
|---|---|---|---|
| Mouse | | | |
| TLR1 | CATTCCTGAGGTCCCTGCTA | GAAGAACTCAGGCGAGCAGA | NM_030682 |
| TLR2 | ACCAAGATCCAGAAGAGCCA | CATCACCGGTCAGAAAACAA | NM_011905 |
| TLR3 | ATAGGGACAAAAGTCCCCCA | ATGATACAGGGATTGCACCC | NM_126166 |
| TLR4 | TGTCATCAGGGACTTTGCTG | TGTTCTTCTCCTGCCTGACA | NM_021297 |
| TLR6 | TCTTGGCTCATGTTGCAGAG | GAGAAAGAAAATGGTACCGTCAG | NM_011604 |
| TLR7 | TGTCTCTTCCGTGTCCACAT | GGATGATCCTGGCCTATCTC | NM_133211 |
| TLR9 | CTGTACCAGGAGGGACAAGG | CAGTTTGTCAGAGGGAGCCT | NM_031178 |
